# A One-Component, Fast-Cure, and Economical Epoxy Resin System Suitable for Liquid Molding of Automotive Composite Parts

**DOI:** 10.3390/ma11050685

**Published:** 2018-04-27

**Authors:** Yiru Wang, Wanshuang Liu, Yiping Qiu, Yi Wei

**Affiliations:** 1 Key Laboratory of Textile Science &Technology, Ministry of Education, College of Textiles, Donghua University, 2999 North Renmin Road, Shanghai 201620, China; yrwang90@sina.com (Y.W.); ypqiu@dhu.edu.cn (Y.Q.); 2 Center for Civil Aviation Composites, Donghua University, 2999 North Renmin Road, Shanghai 201620, China; 3 College of Textiles and Apparel, Quanzhou Normal University, Quanzhou, Fujiang 362000, China

**Keywords:** imidazole, epoxy resin, automotive, carbon fiber, economical composite parts

## Abstract

Imidazole cured epoxy resin systems were evaluated for one-component, fast-curing resins for liquid molding of automotive composite parts according to industry requirements. It was demonstrated that an epoxy resin-1-(cyanoethyl)-2-ethyl-4-methylimidazol(EP-1C2E4MIM) system would cure in a few minutes at 120 °C, while exhibiting acceptable pot life, viscosity profiles, and low water absorption. Moreover, this system yielded high *T*_g_ parts with mechanical properties similar to the amine-epoxy systems, which are the mainstream two-component epoxy resin systems for automobiles.

## 1. Introduction

Owing to the high strength-to-weight ratio and specific stiffness, carbon fiber-reinforced plastics (CFRPs) are increasingly used in a variety of applications that require high-performance and light weight [1,2,3,4,5,6]. CFRPs are regarded as ideal substitutes for metals for the manufacturing of new-generation electric or hybrid car body and parts [7,8]. Likewise, the weight content of CFRPs in Boeing 787 Dreamliner and Airbus A350 has reached over 50% [9]. For modern high-performance CFRPs, epoxy resins are the most widely utilized thermosetting matrix due to their superior mechanical performance, dimensional stability, adhesion, and heat and chemical resistance [10,11,12,13]. Different from aerospace applications, the successful implementation of CFRPs in automotive mass production requires a much higher rate of production and throughput, which sets a significantly shorter time limit for the part cure cycle. The state-of-the-art fast curing epoxy formulations for CFRPs typically includes epoxy resin and hardener (amines or anhydrides) which are kitted as a two-component system, owing to their extremely limited shelf life after being mixed prior to use [14,15]. Compared with two-component epoxy systems, a one-component epoxy resin takes away the hassle of on-site weighting, blending, and de-gassing, especially for plants that have few or no special automation in their infusion process [16,17].

Many strategies have been executed to develop one-component epoxy resins with workable pot life and viscosity profiles [18]. One of the approaches is to take advantage of the dissolubility difference between the epoxy and amine hardener [19,20]. A typical example is the epoxy-dicyandiamide system, which has already been commercialized. However, this type of epoxy system has limitations of high viscosity and relatively long curing time due to the usage of solid hardeners [21]. Chemical blocking is another effective approach to improve the latency of hardeners, for example through the conversion of amines into ketimine derivatives. The modified amines show low curing efficiency because the cross-linking depends on the hydrolysis of imine groups to generate amines [22]. Recently, a series of protected N-heterocyclic carbenes with thermal responsiveness were synthesized and used as a latent catalyst for an epoxy-anhydride mixture. The resulting epoxy systems were fast-curing while still remaining processable from several days to weeks at room temperature. However, these carbene catalysts involved rigorous synthesis procedures and had to be stored in an inert condition [23]. Therefore, the development of one-component epoxy systems with the combination of fast cure, good processability, and economic fabrication is of particular importance.

Imidazoles are a class of epoxy hardener [24,25,26]. Because these hardeners initiate the curing reaction through nucleophilic and anionic polymerization processes, they have high catalytic and cross-linking efficiency. Therefore, only a low level is needed to achieve a high *T*_g_ of the cured epoxy resin, while also maintaining the low cost of the total system. In general, imidazoles have intrinsic high curing reactivity towards epoxy resins, and the curing behaviors can be facilely tailored by introducing various substituent groups on the imidazole rings [27,28]. So far, a large number of imidazole hardeners have been designed and commercialized, providing a generous toolbox to develop one-component and fast-curing epoxy systems [29,30,31]. In this work, we report an epoxy system (EP-1C2E4MIM) based on diglycidyl ether of bisphenol A (DGEBA) and 1-(2-cyanoethyl)-2-ethyl-4-methylimidazole (1C2E4MIM). The inductive effect of the electron-withdrawing cyano group distinctly increases the latency of 1C2E4MIM without sacrificing the curing rate. The rheological, thermal, and mechanical properties of the EP-1C2E4MIM system were evaluated in detail. CFRPs were fabricated by vacuum-assisted resin infusion (VARI) process using this epoxy system as a matrix. The mechanical properties of the resulting composite, including tensile, flexural, compressive, and interlaminar shear properties, were studied.

## 2. Materials and Methods

### 2.1. Materials

DGEBA (E-51) with an epoxy equivalent weight of 182~192 was supplied by Nan Ya Plastic Co. (Zhongshan, China). 2-ethyl-4-methylimidazole (2E4MIM, 99%) and 1C2E4MIM (99%) were purchased from HWRK Chem. Co., Ltd. (Beijing, China). A commercial grade amine-epoxy system 10-min fast-cure epoxy resin 3585/5003-1 was obtained from Wells Advanced Materials (WAM 3585/5003-1) Co., Ltd. (Shanghai, China) as a benchmark. T700 3K carbon fiber plain weave fabric was supplied by Zhongfu Shenying Carbon Fiber Co., Ltd. (Lianyugang, China).

### 2.2. Characterizations

The rheological properties and curing behaviors of the uncured epoxy resins were investigated using a dynamic shear rheometer (DSR) and a differential scanning calorimeter (DSC). The thermo-mechanical properties of the cured epoxy resins were studied by dynamic mechanical analysis (DMA). The mechanical properties of the cured epoxy resins, including fracture toughness, tensile, and flexural properties, were tested according to ASTM standards. For carbon fiber composite laminates, the tensile, flexural, compressive, and interlaminar shear properties were tested according to corresponding ASTM standards.

The viscosity of the epoxy resin was measured by DSR (Haak MARS60) with parallel plates, Φ = 40 mm, a gap of 1 mm, and 1.592 Hz.

The pot life for the epoxy resin at ambient temperature and the gelation times were determined by DSR. The pot life was determined as the time at which the viscosity reached 1000 Pa·s during ambient storage at 23 ± 2 °C.

The cure time of the epoxy resin was determined by DSC (NETZSCH DSC214). Isothermal DSC was performed by rapidly ramping the sample temperature from 0 to 120 °C, with the rate of 40 °C/min, and holding for various periods of time. Dynamic scans at 10 °C/min over a temperature range from 25 to 220 °C were used to determine the total heat of the reaction.

The dynamic thermal mechanical properties were tested on a DMA (Q-800, TA Instruments, New Castle, DE, USA) with a dual cantilever at a heating rate of 2 °C/min from 30 to 250 °C, and a frequency of 1 Hz.

**Figure 1:**
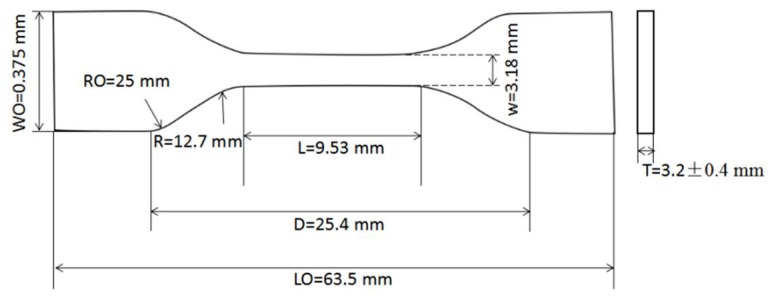
The sample size for tensile test.

**Figure 2:**
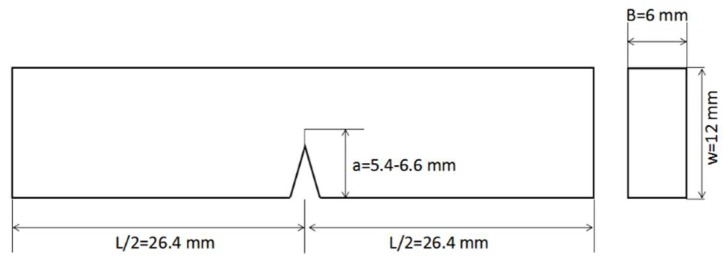
The sample size for the fracture toughness test.

The mechanical tests for neat resins and composites were carried out using a universal testing machine (Instron Model 2360) with a 30-KN load cell. The tensile and flexural tests for neat resins were performed according to ASTM D 638 and D 790, respectively [32,33]. The tensile test sample exhibited a dumbbell shape, as shown in Figure 1. In the figure, W is the width of the narrow section, L is the length of the narrow section, WO is the width overall, and LO is the length overall. D is the distance between grips. R is the radius of fillet. RO is the outer radius. T is the thickness. The tensile test was performed at a crosshead speed of 2 mm/min. The strain was obtained using an electric resistance strain gage. The dimensions of the flexural test sample were 70 mm × 12.7 mm × 3 mm. The flexural test was performed with a span of 56 mm at a crosshead speed of 2 mm/min. The fracture toughness was tested according to ASTM D 5045 [34], and the sample size is shown in Figure 2. The fracture toughness test was performed with a span of 48 mm at a crosshead speed of 10 mm/min. At least six valid specimens were measured for each mechanical test.

The mechanical properties of the carbon fiber composite were also tested according to ASTM standards. The tensile test was performed according to ASTM D 3039 [35] with a sample size of 165 mm × 12.7 mm × 2 mm. The flexural test was performed according to ASTM D 790 with a sample size of 250 mm × 12.7 mm × 4 mm. The compressive test was performed according to ASTM D 3410 [36] with a sample size of 25.2 mm × 12.7 mm × 12.7 mm. The interlaminar shear property was measured by short beam shear test according to ASTM D 2344 [37] with a sample size of 40 mm × 12 mm × 6 mm. The crosshead speed for all of the tests was 2 mm/min and at least six valid specimens were measured for each mechanical test.

The hot-wet properties were characterized by water absorption, hot-wet glass transition temperature (HW-*T*_g_), and hot-wet flexural properties. Water absorption was measured by putting the neat resin sample into a deionized water heat bath of 70 °C and weighing the sample every few days until a constant weight was reached. The moisture absorption was calculated by Equation (1), as follows:

(1) $$H=\frac{G_{i}-G_{0}}{G_{0}}$$

where *G*_0_ is the initial weight of the coupon, and *G*_i_ is the coupon’s weight on the *i*-th day.

HW-*T*_g_ was measured using DMA at a heating rate of 2 °C/min immediately after the neat resin coupon was boiled in water for 48 h.

HW-flexural test coupons were also prepared by boiling in water for 48 h, then immediately tested on the Instron 2360.

### 2.3. Sample Preparation

#### 2.3.1. Preparation of Neat Resin Test Coupons

A mixture of the epoxy resin and imidazole was de-gassed in vacuum at 45 °C for about 1 h. The resulting mixture was heated up to 70 °C, then casted into a preheated aluminum mold coated with release agents, de-gassed for 15 min at 45 °C, and cured for 30 min at 120 °C. The mold was removed from the oven and allowed to cool gradually to room temperature. All samples were polished with a sandpaper (P 7000) to reduce the defects on sample’s surfaces. The benchmark epoxy resin 3585/5003-1 and the hardener were mixed at room temperature and de-gassed for 20 min at room temperature, then casted into a preheated aluminum mold coated with release agents and cured for 30 min at 120 °C.

#### 2.3.2. Preparation of the Carbon Fiber Composite Laminates

A carbon fabric-reinforced epoxy resin laminate was chosen as the composite material becaus of its widespread use in automotive and aeronautical industries. The carbon fabric-reinforced composite coupons were prepared via the vacuum-assisted resin infusion process (VARI). Eight plies of plain weave carbon fabric and one layer of peel ply (30 cm × 30 cm) were laid on a glass plate mold. The de-gassed resin was preheated to 70 °C and infused into the carbon fiber preform, followed by curing for 30 min at 120 °C.

## 3. Results and Discussion

### 3.1. Comparison of the Two Imidazoles

**Figure 3:**
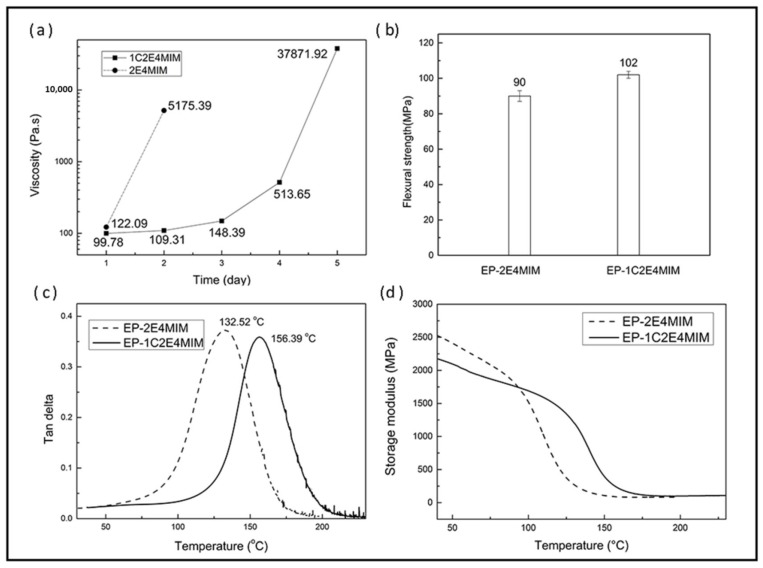
Differences of the two imidazole cured epoxy resins as measured by: (**a**) viscosity change as a function of time at ambient condition; (**b**) neat resin flexural strength; (**c**) Tan δ; and (**d**) storage modulus from dynamic mechanical analysis (DMA).

It is well established that the reactivity of imidazole hardeners depends heavily on their chemical structures. Herein, we selected imidazole compounds with and without an electron-withdrawing group; 1C2E4MIM and 2E4MIM, respectively. The latency of EP-1C2E4MIM and EP-2E4MIM was investigated by measuring the viscosity change with time at 25 °C. As shown in Figure 3a, the viscosity of EP-2E4MIM increased from 122.1 to 5175.4 Pa.s in one day. In contrast, the viscosity increment for EP-1C2E4MIM was relatively slow, and significant viscosity increase was observed on the third day. Apparently the EP-1C2E4MIM showed higher latency, namely a longer pot life. This is attributed to the cyano group on 1C2E4MIM, a strong electron-withdrawing group, that can decrease the electron density of the imidazole ring, leading to the lower reactivity of 1C2E4MIM. Except for the electron effects, the steric effect brought by the cyano group may also decrease the reactivity of 1C2E4MIM. In addition to the more favorable thermal latency, the imidazole with the cyano group had higher flexural strength and glass transition temperature, as shown in Figure 3b–d. Primarily due to the unacceptable pot life, 2E4MIM was dropped from further study in this work.

### 3.2. Evaluation of Different Levels of 1C2E4MIM

#### 3.2.1. Curing Behaviors

The curing behaviors of the EP-1C2E4MIM system were investigated by DSC using isothermal and non-isothermal modes (Table 1). As shown in Table 1, increasing the level of 1C2E4MIM did not affect the onset reaction temperature (*T*_i_), which was determined by the activation energy of the imidazole. However, when the 1C2E4MIM level was increased, the temperature of the maximum exothermic temperature (*T*_max_) showed a slight reduction, while the total enthalpy (ΔH) showed a significant increase. This indicated that the cross-linking reaction was driven to a higher level of completion when the level of 1C2E4MIM was increased from 2 wt % to 6 wt %. From the practical point of view the 1C2E4MIM level of 6 wt % was already too high, because its heat of reaction (ΔH) reached 441 J/g which could induce unexpected intense exothermic reactions and be considered risky in industry. As a result, 1C2E4MIM levels higher than 6 wt % were not further tested.

The results of isothermal DSC at 120 °C also showed that the cross-linking reaction rates increased with increasing 1C2E4MIM level. Table 1 lists the *T*_g_ of the neat resin measured by DMA, along with different levels of 1C2E4MIM, which served as a confirmation of the isothermal DSC results. The data showed that resins with 4 wt % and 6 wt % 1C2E4MIM had their *T*_g_ plateaued, indicating that the resins had reached a complete degree of cure within 15 min. However, that with 2 wt % had a much lower value of *T*_g_, indicating that the cross-linking level was far from adequate.

#### 3.2.2. Thermo-Mechanical Properties

**Figure 4:**
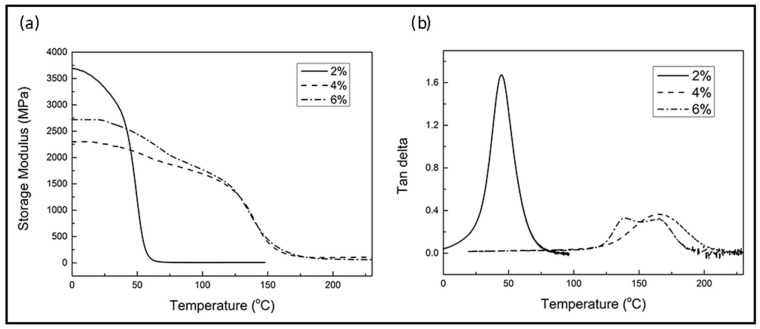
DMA results of the neat resin with different levels of 1C2E4MIM: (**a**) storage modulus; (**b**) Tan δ.

The thermal mechanical properties of the cured neat resin were characterized by DMA. Figure 4a,b show the storage modulus and the Tan δ, respectively, from which the *T*_g_ of the neat resin can be determined. It is well understood that the *T*_g_ of a thermoset resin is mainly affected by intra-molecular stiffness (i.e., molecular rigidity) and cross-linking density [5,30,31]. Therefore, for the same thermoset resin system, a higher *T*_g_ would indicate a higher level of cross-linking. In this case it shows that 2 wt % 1C2E4MIM does not yield adequate cross-linking density and 6 wt % does not provide additional benefit in raising *T*_g_ further compared to that of 4 wt %. 

Perhaps the more significant insight taken from Figure 4 is the appearance of the twin glass transition temperatures at 6 wt % 1C2E4MIM, particularly evident in Figure 4b. It can be seen in Figure 2 that for 1C2E4MIM levels below 4 wt %, the resin *T*_g_ increased as the 1C2E4MIM level increased, indicating an increased cross-linking density. Similarly, their Tan δ curves have only one peak and no relaxations of storage modulus at low temperature, pointing to a homogeneously cured resin. However, when the 1C2E4MIM concentration was further increased beyond 4 wt %, the excessive amount of 1C2E4MIM would induce side reactions, causing phase separation and resulting in two separate glass transition temperatures; one of them was the same as that at 4 wt % (165.6 °C), and the other was lower (137.1 °C).

Although it was unclear what the mechanisms were that caused the side reactions to start to produce molecules or networks exhibiting lower *T*_g_ at that 1C2E4MIM level, it was clear that 6 wt % was not a favorable level based on the intense heat release as shown by DSC results as well as the lowering of the overall *T*_g_ as shown by the DMA. This was the primary reason for selecting 4 wt % as the 1C2E4MIM level for further composite evaluation.

#### 3.2.3. Neat Resin Mechanical Properties

**Figure 5:**
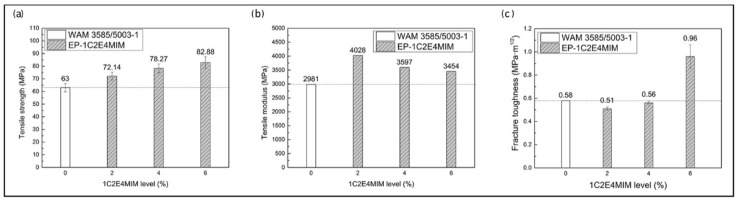
Mechanical properties of neat resins with different levels of 1C2E4MIM, compared to the benchmark resin: (**a**) tensile strength; (**b**) tensile modulus; (**c**) fracture toughness.

To investigate the mechanical properties of the cured epoxy neat resins with different levels of 1C2E4MIM, tensile and fracture toughness tests were performed according to ASTM standards. Meanwhile, a commercial fast-curing epoxy resin, 3585/5003-1, was used as the benchmark and tested side by side. As shown in Figure 5a,b, the tensile strength and modulus of the benchmark resin were 63 and 2981 MPa, respectively. In comparison, the three epoxy resins with different 1C2E4MIM levels showed 14–32% higher tensile strength and 16–35% larger tensile modulus, respectively. The fracture toughness, measured as the critical stress intensity factor (*K*_IC_), is shown in Figure 5c. The *K*_IC_ values of the neat resins with 2 and 4 w t% 1C2E4MIM were 0.51 and 0.56 MPa·m^1/2^, similar to that of the benchmark resin. It was obvious that the neat resin with 6 wt % 1C2E4MIM showed the highest *K*_IC_, which was about 42% higher than that of the epoxy resin with 4 wt % 1C2E4MIM and about 40% higher than that of the benchmark resin. This distinctive high toughness of the 6 wt % 1C2E4MIM sample might be due to the two-*T*_g_/two-phase structure discussed above, for the portion with a lower *T*_g_ could potentially provide some additional toughening. However, the high toughness achieved by 6 wt % 1C2E4MIM could not overweigh its problems with excessive exothermal heat. Additionally, in considering the cost of 1C2E4MIM at high levels, 4 wt % 1C2E4MIM remained the level of choice for subsequent tests.

### 3.3. Evaluation of Resin and Composite with 4 wt % 1C2E4MIM

#### 3.3.1. Shelf Life

**Figure 6:**
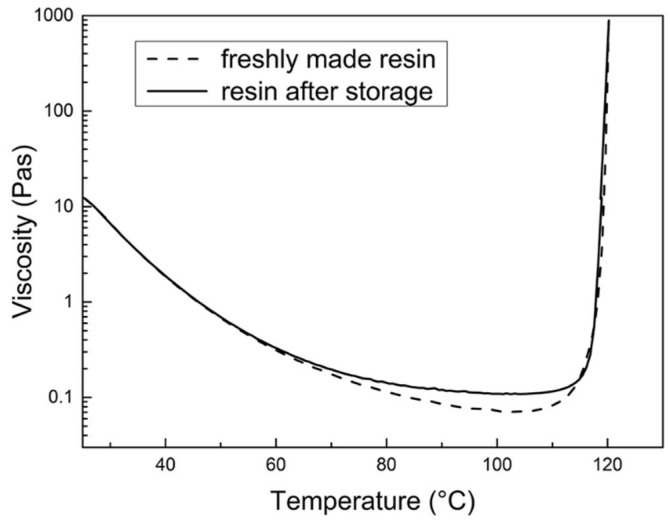
Dynamic shear rheometer (DSR) temperature scan of resins: (**a**) freshly made resin; (**b**) resin after storage at −18 °C for 12 months.

Because the EP-1C2E4MIM resin system evaluated in this work was a fast cure one, it was therefore highly reactive with a potentially limited shelf life. If the shelf life is too short, it would not be suitable for industrial use. For this reason the resin was stored under industry standard storage conditions, at −18 °C, for 12 months and its cure profile was measured by a viscosity-temperature scan using DSR. As can be seen from the results in Figure 6, the viscosity profile of the stored resin remained largely the same compared to the freshly made one, indicating its excellent storage stability and shelf life.

#### 3.3.2. Water Absorption and Hot-Wet Properties

Water resistance is important for composite resins because the absorbed water may cause plasticizing effects, leading to the reduction of *T*_g_ and deterioration of mechanical properties. The water absorption measured by weight gain at 70 °C as a function of time using the neat resin with 4 wt % 1C2E4MIM is shown in Figure 5a, where the water absorption showed a rapid increase within the first 20 days, and then reached a saturated level after 40 days. However, this saturation level, about 1.62 wt %, was much lower than that of HexFlow RTM6 2.42 wt %, which is an extensively used commercial aerospace liquid molding epoxy resin. In addition, the water absorption rate of RTM6 was also higher [32].

**Figure 7:**
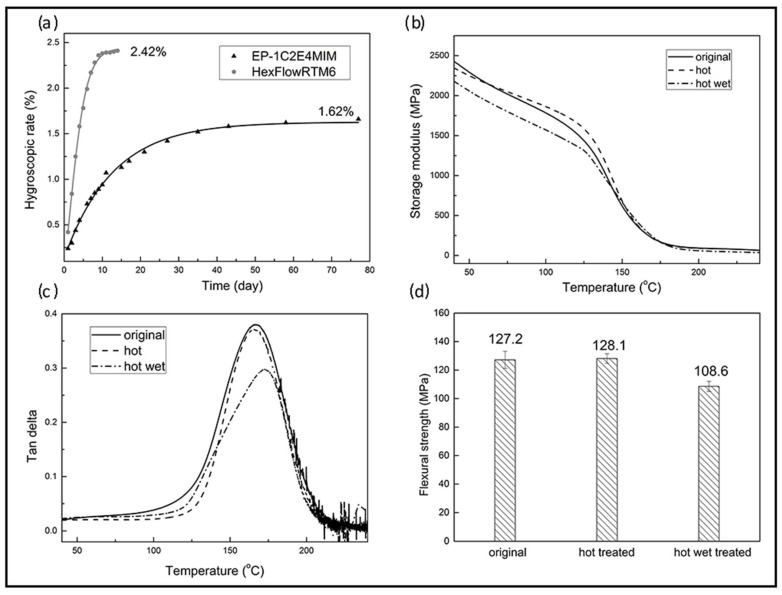
Properties of neat resins: (**a**) the Boltzmann fit of the hygroscopic rate curve of water absorption at 70 °C for 80 days; (**b**) storage modulus; (**c**) Tan δ; and (**d**) flexural strength of original resin, after “hot” heat treatment, and after “hot-wet” conditioning. All resin coupons were prepared with 4 wt % 1C2E4MIM and cured at 120 °C for 30 min.

The excellent water resistance of this resin was also reflected by the zero *T*_g_ change when placed under the “hot” condition, which subjected the resin to heat aging for 48 h at 100 °C. Moreover, the *T*_g_ did not drop, as would occur in most epoxy resin systems, after being subjected to “hot-wet” conditions, although there was a slight drop in its storage modulus, as shown in Figure 7b,c.

Likewise, the flexural strength of the 1C2E4MIM cured epoxy resin remained almost unchanged after heat aging, as shown in Figure 7d. However, for the epoxy coupon aged under hot-wet conditions, its flexural strength showed a distinct decrease from 127.2 to 108.6 MPa. Currently, the exact mechanism of hot-wet aging for epoxy resins is still unclear. The decrease in flexural strength may be because strength is a defect-controlled property for epoxy resins. During the hot-wet treatment, the absorbed water molecule could induce defects into the epoxy network. However, because the *T*_g_ of epoxy resins is mainly determined by the crosslinking density of epoxy networks instead of defects, it is reasonable that this resin exhibited an almost unchanged *T*_g_ value due to its low water absorption.

#### 3.3.3. Mechanical Properties of Carbon Fabric-Reinforced Composites

**Figure 8:**
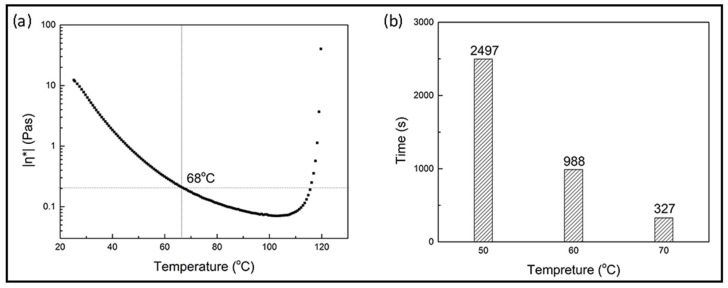
(**a**) DSR temperature scan of EP-1C2E4MIM resin; (**b**) required time for infusion at different temperatures.

The epoxy resin with 4 wt % EP-1C2E4MIM was used as a matrix to fabricate carbon fiber composite test panels. Because the viscosity of the resin at infusing temperature is an important parameter for the VARI process, for it affects the processing time and wettability, the resin viscosity as a function of temperature is shown in Figure 8a. It is shown that the resin’s ambient viscosity was high and not infusible. However, at 68 °C, the viscosity was less than 0.2 Pa.s, which was suitable for VARI processes and thus was used as the reference infusing temperature. When the infusing temperature was lower than 68 °C, the infusing time may be unacceptably long and the fiber might not be properly wetted. In contrast, if the infusing temperature is too high, the resin open time might be compromised. The time required to infuse the same size panels at different temperatures is given in Figure 8b. It is shown that infusion was completed in about 5 min when conducted at 70 °C, which was deemed as favorable in automotive part fabrication. Taking into account of the viscosity and infusion time, the carbon fiber-epoxy composite panels used for mechanical tests were fabricated at the infusion temperature of 70 °C, followed by curing for 30 min at 120 °C.

**Figure 9:**
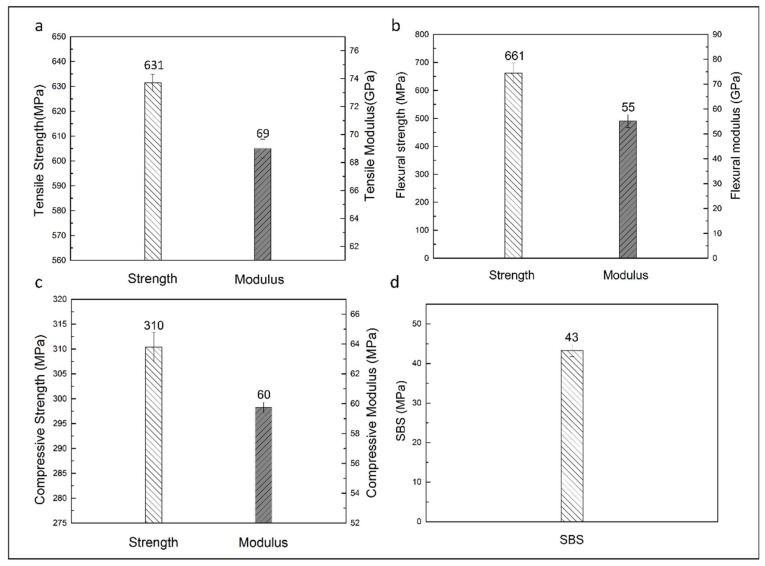
The mechanical properties of carbon fabric composite: (**a**) tensile strength and tensile modulus; (**b**) flexural strength and flexural modulus; (**c**) compressive strength and compressive modulus; (**d**) short beam shear strength. An epoxy system with 4% 1C2E4MIM was used to fabricate the composite laminates.

The mechanical test results of carbon fiber-epoxy composite panels are shown in Figure 9. The average fiber volume fraction (*V*_f_) of the carbon fiber-epoxy composite panels was 68.2%. The data showed that the tensile strength of the composite made with the EP-1C2E4MIM system was 631 MPa, much higher than that made from 6808 epoxy resin (374 MPa) [38]. Employed here as a reference, 6808 epoxy resin is a commercial resin used for automobile part fabrication. The compressive strength is 310 MPa, which is slightly higher than that of the composite with 6808 resin (308 MPa) [38]. The flexural strength and short beam shear strength are 661 MPa and 43 MPa, respectively, which are deemed acceptable. The flexural and short beam shear strengths were not available from 6808 resin for comparison. Overall, these composite mechanical tests demonstrated the good compatibility of the current EP-1C2E4MIM resin with carbon fibers. The resultant composite exhibited excellent mechanical strength, rendering it a potential candidate for the intended high-throughput automotive composite part fabrication.

## 4. Conclusions

Imidazoles 2E4MIM and 1C2E4MIM were investigated as hardeners for fast cure one-component epoxy resins, which could be used in high-throughput automotive composite part production. Compared to 2E4MIM-cured epoxy resins, 1C2E4MIM-cured epoxies had longer pot life as well as higher *T*_g_ and flexural strength when cured at the same conditions, and therefore was deemed more favorable for the intended applications. It was demonstrated that the resins made with 4 wt % 1C2E4MIM loading had a combination of fast cure, reasonable pot life, high *T*_g_, long shelf life at −18 °C, and low moisture uptake, and thus was suitable for liquid molding processes such as VARI. The mechanical properties of the composite made with this resin and a T 700 grade carbon fabric were also tested and shown to be superior to that of a current commercial product.

## Figures and Tables

**Table 1:** Cross-linking characteristics of EP-1C2E4MIM measured by differential scanning calorimetry (DSC) and DMA.

1C2E4MIM (wt %)	*T*_i_^(1)^ (^o^C)	*T*_max_^(2)^ (^o^C)	ΔH ^(3)^ (J/g)	Time to Reach Complete Cure at 120 °C (min)	*T*_g_^(4)^ (°C)
2%	135.1	155.1	128.8	27	71.6
4%	135.0	150.4	353.3	15	166.4
6%	134.3	147.5	441.1	8	137.1, 165.6

^(1)^ Onset temperature; ^(2)^ Peak temperature; ^(3)^ Enthalpy of cure reaction; ^(4)^ *T*_g_ by DMA.
